# Clinical predictors associated with full remission versus episode of major depressive disorder outpatients: the experience at a teaching hospital in Taiwan

**DOI:** 10.1186/s12888-014-0273-0

**Published:** 2014-09-24

**Authors:** Mei-Yu Yeh, Yu Lee, Su-Ching Sung, Tao-Hsin Tung

**Affiliations:** Graduate Institute of Health Care, Chang Gung University of Science and Technology, Taoyuan, Taiwan; Department of Psychiatry, Kaohsiung Chang Gung Memorial Hospital, Kaohsiung, Taiwan; Department of Medical Research and Education, Cheng-Hsin General Hospital, Taipei, Taiwan; Faculty of Public Health, School of Medicine, Fu-Jen Catholic University, Taipei, Taiwan

**Keywords:** Episode, Major depressive disorder, Remission

## Abstract

**Background:**

When depressed patients are in remission, the clinical characteristics indicate that they are able to participate in social activities more regularly, and their impairment in daily functioning is improved. The present study examines the clinical characteristics associated with one- and two month clinical response in outpatients with Major Depressive Disorder (MDD) in Taiwan.

**Methods:**

A total of 160 outpatients were initially recruited from the medical centre in Taiwan. Of these participants, 151 MDD patients completed the baseline-assessment interview, 111 were interviewed and assessed again 4 weeks later, and 78 completed the final interview and assessment 8 weeks later. In the present study, asymptomatic was defined as scoring ≤ 7 on the Hamilton Depression Rating Scale (HAM-D); partially symptomatic was defined as scoring 8–14; fully symptomatic was defined as scoring ≥15. Finally, asymptomatic, partially symptomatic, and fully symptomatic were defined in patients with MDD respectively as in full remission, in persistent depressive symptom, and in episode.

**Results:**

Of the remaining 78 patients, a total of 21 (26.9%) were in full remission, 35 (44.9%) were in persistent depressive symptom, and 22 (28.2%) were in episode. Patients in full remission were older (p = 0.03), exhibited greater psychosocial functioning, (p < 0.001), held more-positive beliefs regarding antidepressant medication (p = 0.03), had higher self-efficacy (p = 0.001), and scored lower for neuroticism (p = 0.003), as compared to patients in episode. Younger patients were more prevalent in persistent depression. Repeated-measures ANOVA revealed that differences in four factors (psychosocial functioning, beliefs regarding antidepressant medication, self-efficacy in managing and preventing depression, and neuroticism) were significantly different between full remission and episode. Episode was significantly associated with psychosocial-functioning impairment (OR = 1.12, 95% CI: 1.00-1.26) and poorer self-efficacy (OR = 0.91, 95% CI: 0.82-1.00).

**Conclusions:**

Our findings identify significant factors of full remission, persistent depressive symptom, and episode. We highlight the importance of enhancing patients’ psychosocial functioning and self-efficacy until achieving full remission. Suggestions are provided for clinical health-care management services in Taiwan.

## Background

Depression is a severe illness. It is a recurring, disabling condition with high costs, such as extensive pharmacotherapy and decreased personal productivity, profoundly affecting patients’ lives [1]. A recent study shows a low remission rate for Major Depressive Disorder (MDD) patients after taking antidepressant medication for 3 to 12 months in primary care practice: 28.3% according to the general practitioners and 17.1% according to the patients [2]. Another study showed that approximately two thirds of patients failed to achieve full remission, and only 26% reached full remission after 6 months with acute antidepressant-medication treatment [3].

Remission, featuring a Hamilton Depression Rating Scale (HAM-D) score of seven or less, means that a patient has been in a symptom-free state and has returned to normal levels of psychosocial functioning [4-6]. Treating depressed patients until they reach a state of remission confers the benefit of reduced risk of relapse or recurrence [2,7]. The consequences of achieving full symptom resolution could be greater psychosocial functioning and quality of life for the patients with major depression [2,8]. The social- and occupational functioning and quality of life for patients diagnosed with depression appear to be negatively influenced by their illness [3,4]. However, when depressed patients are in remission, the clinical characteristics indicate that they are able to participate in social activities more regularly, and their impairment in daily functioning is improved. In addition, they are more confident in managing their depression and preventing a relapse of depression; are more likely to express positive beliefs regarding antidepressant medication; and reveal a lower level of neurosis [4,9]. Self-efficacy is a significant characteristic of remission in regards to controlling or preventing future episodes [10,11]. Promoting self-efficacy for managing depression (e.g. regular keeping track of depressive symptoms using a depression symptom inventory, engaging in pleasant activities and engaging in social activities) increased the likelihood of performing several behaviors related to controlling depression [11].

Katon et al. [12] found that relapse-prevention programs were strongly associated with improvement of beliefs regarding antidepressant medication, as well as a significant decrease in depression symptoms. A lower adherence to antidepressant medication is consistently associated with a higher rate of relapse [11,13]. If the patient remains asymptomatic while taking medication, she will have more-positive beliefs regarding medication [12]. Neuroticism is a broad, higher-order personality factor that refers to emotional instability and vulnerability to stress [14]. Accordingly, it might be expected that levels of neuroticism could be used to predict depression, either as a main effect or stemming from stressful life events (such as financial failures, failure to resolve interpersonal conflicts or problems, etc.) [14].

The research results of Romera et al. [3] clearly emphasize the importance of treating the patient until achieving full remission as opposed to partial remission, because the “persistence of partial remission has been associated with an increased risk of relapse, poorer quality of life, and disability”. Full remission is defined as the individual being asymptomatic for 2 weeks to 6 months, a symptom-free state with a return to pre-morbid functioning, i.e. normal levels of psychosocial functioning [9]. Ciudad [15] reported that in the clinical course of major depression, early response within 6 weeks and remission was strongly associated with good outcomes at a 12-month follow-up. Kocsis [16] indicated remission as the greatest predictor of continued remission during 2 years of follow-up. Therefore, the question arises as to how to facilitate remission or recovery from depression. The objective of this study was to use the HAM-D to examine clinical factors associated with full remission and episodes of MDD.

## Methods

### Participants

All participants were selected and interviewed by their psychiatrist in the outpatient-services clinic in the department of psychiatry of a medical centre in southern Taiwan. Participants were diagnosed with MDD by 11 psychiatrists using the DSM-IV criteria, but those with personality disorders, bipolar disorder, substance-induced depression, and psychosis with depression were excluded.

**Figure 1:**
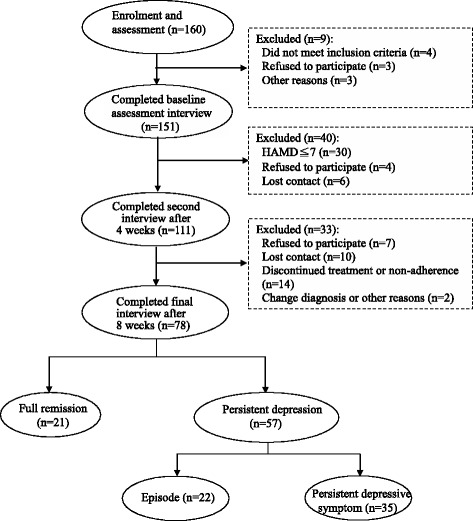
**The recruitment procedure of outpatients with MDD.**

A total of 160 outpatients were initially recruited from the medical centre in Taiwan. Of these participants, 151 completed the baseline interview, 111 subjects completed the second interview after 4 weeks, and 78 outpatients completed the third interview after 8 weeks. The recruitment procedure is shown in Figure 1.

### Ethical considerations

The recruitment protocol and the study protocol were approved by the institutional review boards of the Kaohsiung Chang Gung Memorial Hospital in Taiwan. Before contacting participants, researchers communicated and explained the research procedure and recruiting criteria to psychiatrists, and emphasised that subjects’ responses would be anonymous and confidential, unless subjects agreed to share the results with their physicians.

### Measures

#### Hamilton depression rating scale (HAM-D)

Since the HAM-D was published in 1960, it has been used as an instrument for assessing the severity of depression and response to therapy in clinical research. It is the most popular measure to evaluate remission [6,17]. Keller [4] indicated that HAM-D ≤7 is the definition of asymptomatic, and an asymptomatic state continuing for two weeks to six months was defined as reaching a state of remission. Fully symptomatic was defined as scoring ≥15. Being in episode was defined as remaining fully symptomatic for more than two weeks.

In the present study, the HAM-D evaluations were available for baseline interviews, the second interview after 4 weeks, and the third interview after 8 weeks. Patients who were asymptomatic at the baseline interview we’re not included in this study. Being in full remission was defined as scoring ≤7 at both 4 weeks and 8 weeks. Being in episode was defined as remaining fully symptomatic at both 4 weeks and 8 weeks. Being in persistent depressive symptom was defined as being neither in full remission (scoring ≤7 at 4 weeks and 8 weeks), nor in episode (scoring ≥15 at 4 weeks and 8 weeks), but at least once partially or fully symptomatic (Table 1).

**Table 1:** **Operational definition of full remission and persistent depression**

		**Hamilton rating scale in this study**			
		**Definition**	**Baseline**	**After 4 weeks**	**After 8 weeks**
Full remission		Asymptomatic at ≥4 weeks	Score >7	Asymptomatic (Score ≤ 7)	Asymptomatic (Score ≤ 7)
Persistent depression	Persistent depressive symptom	Partially or fully symptomatic at ≥4 weeks	Score >7	At least once partially (Score 8–14) or fully symptomatic (Score ≥15)	
	Episode	Fully symptomatic at ≥4 weeks	Score >7	Fully symptomatic (Score ≥15)	Fully Symptomatic (Score ≥15)

#### Psychosocial functioning

The three psychosocial-functioning factors with regard to health’s impact level in daily life, adapted from the National Health Interview Survey [10], are: occupational function, daily life with family, and leisure and social activities. Each psychosocial functioning factor is scored from 0 to 10, with higher scores representing greater impairment of psychosocial functioning.

#### Medication belief scale

The 12-item Medication Beliefs Scale, in a 5-point Likert format, is a reliable and valid instrument to measure beliefs about medication, with responses ranging from 1 (Strongly Disagree) to 5 (Strongly Agree). Items include, for example: “drugs for anxiety or depression are not the answer to problems in one’s life”; “prescription drugs are a crutch”; and “I should be able to get by without using these kinds of drugs”. The 12 items are scored to provide a measure of overall confidence in antidepressant medication ranging from 0 to 5, with higher scores representing greater confidence in antidepressant medication. The original-scale Cronbach’s alpha for this 12-item score was 0.81, following which all 12 items were loaded into one factor in the Principle Component Analysis [12].

#### Self-efficacy for managing depression

Self-efficacy was assessed using a 6-item scale which consisted of 2 global questions on managing and preventing depression, and 4 items for specific behavioral issues [11]. Items included: 1) How confident are you in your ability to overcome or control a bout of depression? 2) How confident are you in your ability to prevent depression from returning once you are better? 3) How confident are you in your ability to recognize early on when you are starting to get depressed? The 6 items are scored from 0 to 10, providing a measure of overall self-efficacy, with higher scores representing greater confidence of self-efficacy for managing depression.

#### Personality traits

The 60-item NEO Personality Inventory isolates five general personality traits: neuroticism, extroversion, openness to experience, agreeableness, and conscientiousness [18]. The 60 items, with Likert-scale responses ranging from 1 (Strongly Disagree) to 5 (Strongly Agree), are scored to provide a measure of the traits, with higher scores representing higher neuroticism.

### Procedures

All processes involving patients were coordinated with an outpatient-treatment clinic. The research instruments were back-translated into Chinese by the researchers, and pre-tested with twenty depressed patients. All participants were referred by their psychiatrist. The researchers explained to each participant that the main purpose of the study was to identify treatment strategies for primary care settings. It was emphasized that all processes were anonymous and voluntary; the risks and benefits of participation were explained, including patients’ right to refuse to participate without jeopardising treatment. Subsequent to this explanation, patients who decided to participate in the interviews signed an informed-consent form. All patients were treated by psychiatrists and were provided with pharmacotherapy and clinical management, including discussing the effects and side-effects of medication, encouraging the patient to comply with the medication regimen, and providing practical and emotional support. Following this, data was collected through one-on-one interviews with the researchers and self-reporting of participants on questionnaires.

In this study, in order to reduce participant dropout, the researcher visited each patient at the psychiatric clinic, and reconfirmed participation by telephone before the follow-up interviews at 4 weeks and 8 weeks. Also, the participants received NT$200 dollars after each of their interviews were completed.

### Statistics analysis

Statistical analysis was performed using the SAS 9.1 software package. In the univariate analysis, independent sample t-test and chi-square test were performed to identify socio-demographic factors associated with full remission, persistent depression, and episode, as well as variables shared between them. Repeated-measures ANOVA was used to estimate the time trends of variables shared between full remission and episode. Finally, multiple-logistic regression was used to investigate the independent variables related to full remission, persistent depression, and episode. The odds ratio and a 95% confidence interval were used for categorical and continuous variables. A p-value of <0.05 was considered statistically significant.

## Results

There were 151 participants that completed the baseline-assessment interview, 111 of those 151 participants completed the second interview and assessment after 4 weeks, and 78 participants completed the final interview and assessment after 8 weeks. Of the remaining 78 patients, a total of 21 (26.9%) were in full remission, 35 (44.9%) were in persistent depressive symptom, and 22 (28.2%) were in episode (Figure 1).

As shown in Table 2, demographic characteristics, number of episodes, pharmacotherapy, and psychotherapy were similar among patients in full remission, persistent depression, and episode. Patients in full remission were older (p = 0.03; p = 0.01), exhibited greater psychosocial functioning, (p < 0.001), held more-positive beliefs regarding antidepressant medication (p = 0.03; p = 0.02), had higher self-efficacy (p = 0.001), and scored lower for neuroticism (p = 0.003), as compared to patients in persistent depression, and episode (Table 2).

**Table 2:** **Comparison of predictors between full remission, persistent depression, and episode**

		**Full remission (** ***n*** **= 21)**		**Persistent depression (** ***n*** **= 57)**		**Episode (** ***n*** **= 22)**		***P*** ^***a***^	***P*** ^***b***^
Categorical variables*		**n**	**%**	**n**	**%**	**n**	**%**		
Gender	Male	11	52.4	18	31.6	8	36.4	0.29	0.09
	Female	10	47.6	39	68.4	14	63.6		
Education	Junior high school or below	10	47.6	28	49.1	13	59.1	0.65	0.97
	Senior high school	6	28.6	17	29.8	6	27.3		
	College or above	5	23.8	12	21.0	3	13.6		
Fixed family income	No	4	19.0	17	29.8	7	31.8	0.27	0.34
	Yes	17	81.0	40	70.2	15	68.2		
Employment	Unemployed	15	71.4	31	54.4	15	68.2	0.54	0.33
	Employed	6	28.6	26	45.6	7	31.8		
Religion	None	6	28.6	17	29.8	5	22.7	0.51	0.73
	Buddhism or Taoism	11	52.4	34	59.7	15	68.2		
	Others	4	19.0	6	10.5	2	9.1		
Marital Status	Single	2	9.5	7	12.3	3	13.6	0.31	0.16
	Married	16	76.2	38	66.7	12	54.5		
	Other	3	14.3	12	21.0	7	31.8		
Number of previous episodes	<4	5	23.8	9	15.8	1	4.5	0.07	0.41
	≧4	16	76.2	48	84.2	21	95.5		
Pharmaco-therapy	No	3	14.3	19	33.3	4	18.2	0.66	0.08
	Yes	18	85.7	38	66.7	18	81.8		
Psychotherapy	No	14	66.7	47	82.5	16	77.3	0.54	0.21
	Yes	7	33.3	10	17.5	6	22.7		
Hospitalization	0	12	57.1	30	52.6	12	54.4	0.50	0.80
(times)	1	6	28.6	15	26.3	4	18.2		
	≧2	3	14.3	12	21.1	6	27.3		
**Continuous variables†**									
Age (years)		57.1 ± 14.0	48.3 ± 13.8			47.7 ± 13.2		0.03	0.01
Psychosocial functioning		5.9 ± 4.0	15.6 ± 9.4			18.3 ± 9.2		<0.001	<0.001
Beliefs regarding antidepressant medication		44.5 ± 4.7	42.0 ± 4.0			41.3 ± 4.7		0.03	0.02
Self-efficacy		45.6 ± 9.7	36.6 ± 10.7			32.4 ± 10.5		<0.001	0.001
Neuroticism		19.5 ± 5.5	24.1 ± 5.9			25.5 ± 6.9		0.003	0.003

*Categorical variables were compared with *χ* ^*2*^ tests.

†Continuous variables were compared with independent sample *t* tests.

^a^p value for *χ* ^*2*^ tests or t-tests, full remission v.s. episode.

^b^p value for *χ* ^*2*^ tests or t-tests, full remission v.s. persistent depression.

**Figure 2:**
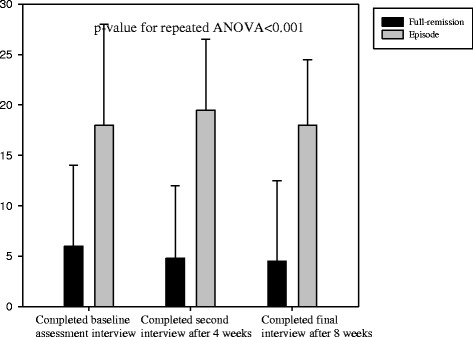
**The time-trend of psychosocial functioning among full-remission and episode subjects.**

**Figure 3:**
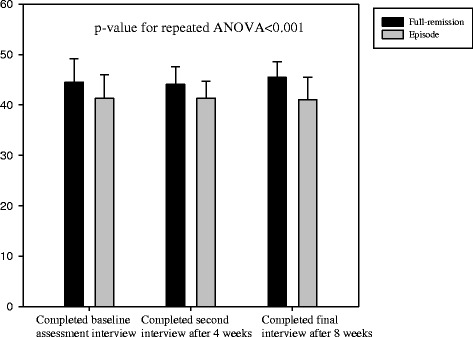
**The time-trend of drug beliefs among full-remission and episode subjects.**

**Figure 4:**
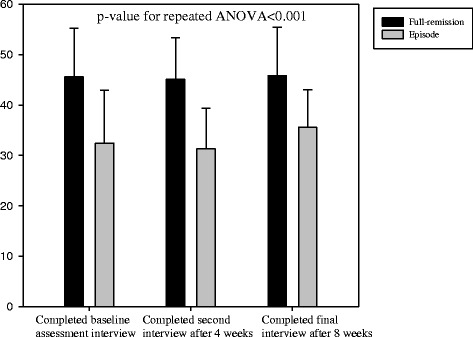
**The time-trend of self-efficacy among full-remission and episode subjects.**

**Figure 5:**
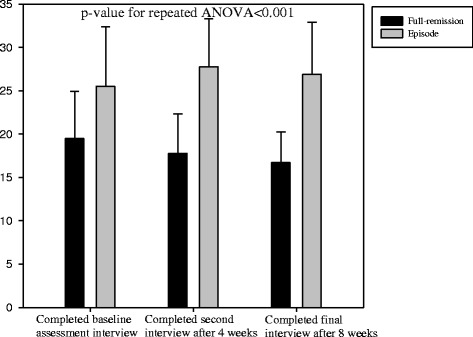
**The time-trend of neuroticism among full-remission and episode subjects.**

In addition, repeated-measures ANOVA revealed that in comparison with patients in episode, those in full remission showed significantly greater psychosocial functioning (p < 0.001), more positive beliefs regarding antidepressant medication (p < 0.001), greater self-efficacy for managing and preventing depression (p < 0.001), and lower levels of neuroticism (p < 0.001) at baseline assessment, after 4 weeks, and after 8 weeks. These are shown in Figures 2, 3, 4 and 5.

The multiple logistic regression models in univariate analyses were used to examine which factors had independent effects on patients in full remission as compared with those in persistent depression, and patients in full remission as compared with those in episode. As shown in Table 3, psychosocial-functioning impairment (OR = 1.11, 95% CI: 1.00-1.22) was the only significant factor for patients in persistent depression. Episode was significantly associated with psychosocial- functioning impairment (OR = 1.12, 95% CI: 1.00-1.26) and poorer self-efficacy (OR = 0.91, 95% CI: 0.82-1.00). After adjustment for confounding factors, age, beliefs regarding antidepressant medication, and neuroticism were not deemed clinically significant related to episode.

**Table 3:** **The multiple logistic regression on the factors associated with persistent depression and episode compared with full remission**

**Variable**	**Full remission (n = 21) vs. persistent depression (n = 57)**					**Full remission (n = 21) vs. episode (n = 22)**				
	**β value**	**SE**	**OR**	**95% CI**	**p-value**	**β value**	**SE**	**OR**	**95% CI**	**p-value**
Age	−0.004	0.03	1.00	0.94-1.05	0.88	−0.02	0.04	0.98	0.91-1.06	0.67
Psychosocial functioning	0.10	0.05	1.11	1.00-1.22	0.04	0.12	0.06	1.12	1.00-1.26	0.04
Beliefs regarding antidepressant medication	−0.02	0.07	0.98	0.85-1.13	0.78	−0.02	0.09	0.98	0.82-1.17	0.84
Self-efficacy	−0.05	0.04	0.95	0.88-1.02	0.13	−0.10	0.05	0.91	0.82-1.00	0.04
Neuroticism	−0.03	0.08	0.98	0.84-1.13	0.74	−0.07	0.10	0.93	0.76-1.14	0.49

## Discussion

Several important findings have emerged from this study. An aspect of this study was evaluation of patients in full remission compared to patients in persistent depression and with those in episode. There are few studies that have evaluated the psychosocial and functional outcomes of depression [3]. Our main finding with multiple logistic regressions was that psychosocial functioning was the main associated factor of full remission compared with both persistent depression and episode. This result was similar to Enns’ study [14], and it was closely linked between the clinical significant factors and functional outcome with depression patients [19]. Functional status is an important indicator in depression, when depressed patients achieve full remission, they be able to restore the premorbid psychosocial function status, and to participant social activities regularly, and confident for managing the depression [8,20,21].

Self-efficacy was another associated factor for patients in full remission of depression compared to patients in episode. Self-efficacy could positively predict improvements in depression-symptom scores over time within individuals [10,11]. In this study, self-efficacy is also a significant characteristic of remission in regards to controlling or preventing future episodes. Clinical professionals need to help MDD patients foster abilities to overcome or control their depression symptoms, screen for warning signs of deepening depression, and improve patients’ confidence for managing their own life and disease. Collaborative care management has been demonstrated to be effective for improving depression outcomes [20].

Previous studies showed that younger patients were more prevalent in persistent depression and episode. In the present study, the mean age of the patients at high risk for persistent depression and episode is around 47 to 48 years old. Similarly, the study of Enns and Cox [14] found that patients around 42 years old had poorer outcomes of major depression as compared with 39.6 years. They indicated that patients who encounter achievement life events (for example, work or financial problems) and interpersonal stressful life events (for example, relationship discord or death of an important person), particularly when they have an avoidant coping style, are at risk for continued depressive symptoms [14]. In this study, however, patients’ age, beliefs regarding antidepressant medication, and neuroticism were significantly associated with persistent depression and episode when compared with full remission in the univariate analysis, but not in the logistic regression. Further study is needed to explore the causal relationship between age, psychosocial functioning, beliefs regarding antidepressant medication, self-efficacy, and neuroticism.

Resolutions of major depression should focus on both symptoms and functional impairments. Depression leads to high levels of unemployment, disabilities, and decreased work performance, and has serious effects on patients, their families, and ultimately the community. Thus, it is important to help patients manage their disease to avoid relapses, improve their quality of life, and decrease associated disabilities [7].

The four factors that this study revealed to significantly improve depression were psychosocial functioning, beliefs regarding antidepressant medication, self-efficacy, and neuroticism. High neuroticism scores have been found to be among the best predictors of persistent depression [18], and neuroticism might be related to stressful life events [16]. Therefore, relapse-prevention programs should include: 1) Rebuilding psychosocial life and functioning, 2) Encouragement of positive beliefs regarding antidepressant medication, 3) Reconstruction of patients’ confidence in managing their depression and preventing relapses, and 4) Management of life events.

### Limitations

Our study has several limitations. This small longitudinal study (n = 151) only assessed clinical characteristics with regard to their predictive values in short-term treatment (2 months) with a limited numbers of visits (in our case, 3 visits), and only 51.7% of the participants completed all three interviews. Any biases associated with data-collecting, and the long-term predictive value of the factors revealed to be significant in this study (psychosocial functioning, beliefs regarding antidepressant medication, self-efficacy, and neuroticism) are yet unknown. Lacking patients’ history of antidepressant intake, we were unable to report on the efficacy and quality of pharmacotherapy treatment (e.g., each participant’s antidepressant therapy dosage and duration prior to our study) or to determine patient- and clinician-related factors that may have influenced whether antidepressants were continued.

We could not ascertain the types of treatment offered beyond pharmacological interventions during the study and the number of psychotherapy or combined-therapy visits, if any, prior to our study. Aside from self-report assessment of the duration of the current depressive episode, we could not ascertain to what extent interpersonal events and responses to depression (that is, coping styles) played in the participants of our study. In the absence of complete patient case records, we were unable to ascertain whether participants experienced more frequent switches into or out of major depression, and whether transient fluctuations influenced depression status in-between each follow-up interval.

### Clinical implications

Our findings have several clinical implications: (1) In light of prior longitudinal studies, some of the predictors highlighted in our short-term study of remission appeared to have potentially long-term predictive values [13,16]. More specifically, while low neuroticism scores were found in our remitted patients, Katon et al. [18] observed that severity of depression and high neuroticism scores were the best predictors of persistent depression. (2) As this is the first investigation of clinical characteristics of remission in a Taiwanese population, it may be enlightening to take into consideration that pharmacological interventions seemed more habitually/conventionally acceptable to Taiwanese outpatients than other forms of professional interventions, such as psychotherapy and/or combined therapy. (3) It would be clinically meaningful to conduct a study on treatment preferences or treatment availability for Taiwanese outpatients with MDD.

## Conclusions

Experts in depression treatment have gained insights into how disabling and costly MDD is and how important it is to treat depressed patients to full remission [1,22,23]. Expanding on previous studies, our findings identify a number of factors correlated to full remission from depression: such as greater psychosocial functioning, more-positive beliefs regarding antidepressant medication, heightened self-efficacy in managing/preventing depression, and lowered neuroticism.
